# Mechanism for CCC DNA Synthesis in Hepadnaviruses

**DOI:** 10.1371/journal.pone.0008093

**Published:** 2009-11-30

**Authors:** Ji A. Sohn, Samuel Litwin, Christoph Seeger

**Affiliations:** Fox Chase Cancer Center, Philadelphia, Pennsylvania, United States of America; Yonsei University, Republic of Korea

## Abstract

Hepadnavirus replication requires the synthesis of a covalently closed circular (CCC) DNA from the relaxed circular (RC) viral genome by an unknown mechanism. CCC DNA formation could require enzymatic activities of the viral reverse transcriptase (RT), or cellular DNA repair enzymes, or both. Physical mapping of the 5′ and 3′ ends of RC DNA and sequence analysis of CCC DNA revealed that CCC DNA synthesis requires the removal of the RT and an RNA oligomer from the 5′ ends of minus and plus strand DNA, respectively, removal of sequences from the terminally redundant minus strand, completion of the less than full-length plus strand, and ligation of the ends. Two models have been proposed that could explain CCC DNA formation. The first (model 1) invokes a role for the RT to catalyze a cleavage-ligation reaction leading to the formation of a unit length minus strand in CCC DNA and a DNA repair reaction for the completion and ligation of plus strand DNA; the second (model 2) predicts that CCC DNA formation depends entirely on cellular DNA repair enzymes. To determine which mechanism is utilized, we developed cell lines expressing duck hepatitis B virus genomes carrying mutations permitting us to follow the fate of viral DNA sequences during their conversion from RC to CCC DNA. Our results demonstrated that the oligomer at the 5′ end of minus strand DNA is completely or at least partially removed prior to CCC DNA synthesis. The results indicated that both RC DNA strands undergo DNA repair reactions carried out by the cellular DNA repair machinery as predicted by model 2. Thus, our study provided the basis for the identification of the cellular components required for CCC DNA formation.

## Introduction

Hepadnaviruses are small DNA viruses that replicate their genomes by reverse transcription of an RNA intermediate [1], [2]. The viral genomes are in a relaxed circular conformation that is stabilized by cohesive overlaps created by the juxtaposition of the 5′ ends of the two DNA strands [3]. Hepadnaviruses are enveloped viruses that primarily infect hepatocytes by a pH-independent pathway that is still incompletely understood. Following uncoating of the viral envelope, core particles are released into the cytoplasm and eventually enter nuclear pores and perhaps the nucleus, disassemble and release RC DNA [4], [5]. Within a few hours after an infection, CCC DNA derived from RC DNA in virions can be detected in nuclei of infected hepatocytes [6], [7]. During early stages of infection, additional CCC DNA is produced from newly synthesized RC DNA present in cytoplasmic core particles by an intracellular amplification pathway [8], [9]. As a consequence of this mechanism, infected cells harbor between 5–30 copies of CCC DNA and remain persistently infected even in the presence of antiviral therapies that inhibit the RT (i.e. ref. [10]).

**Figure 1:**
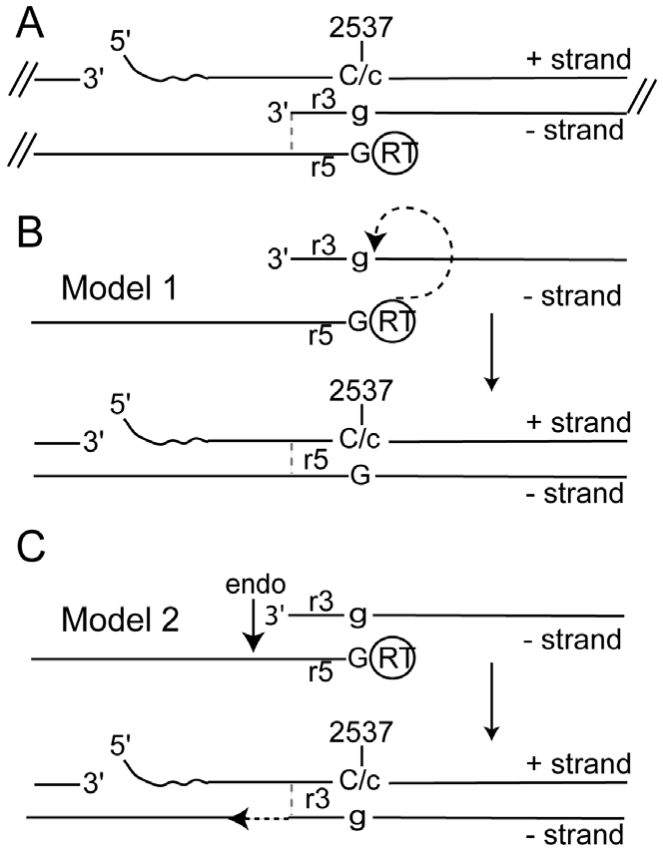
Models for CCC DNA formation. A. The figure shows a segment of the DHBV genome comprising the 5′ and 3′ ends of plus (+) and minus (−) strands in RC DNA. The 5′ ends of the DNA strands are covalently attached to RNA (waved line) and reverse transcriptase (RT). Minus strands bear 9 nucleotide-long terminal repeats, r5 and r3. Position 2537 marks the first nucleotide, dGMP (G) attached to the RT ([27], accession number K01834). The corresponding nucleotides in r3 (g) and plus strand DNA C/c are indicated. Note, dC on plus strands could be derived from G (C) or g (c), depending on the timing of the template switch from r5 to r3 necessary for plus strand synthesis (see text). The vertical bar indicates the positions of the 3′ ends of r3 and r5, respectively. B and C. The figures depict two models (model 1 and 2) for CCC DNA synthesis described in the text. Endo; endonuclease.

CCC DNA synthesis requires the removal of a 18 nucleotide-long RNA primer from the 5′ end of plus strand DNA and the reverse transcriptase from the 5′ end of minus strand DNA [11], [12]. In addition, one or both ends of minus strand DNA have to be trimmed to remove all or some of the sequences in the 9 nucleotide-long terminal redundant r5 and r3 segments. The final step in CCC DNA synthesis is the ligation of the 5′ and 3′ ends of the two DNA strands. (Figure 1A). The exact sequence of events and the enzymatic activities leading to CCC DNA synthesis have not yet been described.

Two models can explain the formation of CCC DNA (Figure 1B,C). The first (model 1) predicts that the reverse transcriptase performs a cleavage-ligation reaction to synthesize the minus strand of CCC DNA, which then could serve as a template for the repair of plus strand DNA. For this reaction, the RT would have to hydrolyze the phosphodiester bond at the 5′ end of the 3′r region and use the released energy for a transesterification reaction resulting in the dissociation of the RT from the 5′ end and the ligation of the two ends of minus strand DNA. A similar biochemical reaction is carried out by the A protein of bacteriophage ΦX174 during rolling circle DNA replication [13]. It has been suggested that an RC DNA form lacking RT at the 5′ end of minus strand DNA might be a precursor for CCC DNA formation essentially as predicted by model 1 [14], [15]. The second model (model 2) predicts that a cellular DNA endonuclease cleaves minus strand DNA downstream of the 5′ end and that a cellular DNA polymerase extends the 3′ end using plus strand DNA as a template followed by the ligation of the free ends. Thus, the second model would occur independently of a viral enzymatic activity and as a consequence would depend entirely on cellular DNA repair enzymes.

The low efficiency of CCC DNA formation in tissue culture cells and the lack of permissive *in vitro* systems to recapitulate the conversion of RC to CCC DNA, hampered efforts to investigate the first step critical in hepadnaviral DNA synthesis. As described in this report, we have exploited information about the priming reaction required for reverse transcription of HBV genomes for a genetic analysis of CCC DNA formation that permitted a distinction between the two models proposed above for CCC DNA synthesis.

## Results and Discussion

### Experimental Strategy

**Figure 2:**
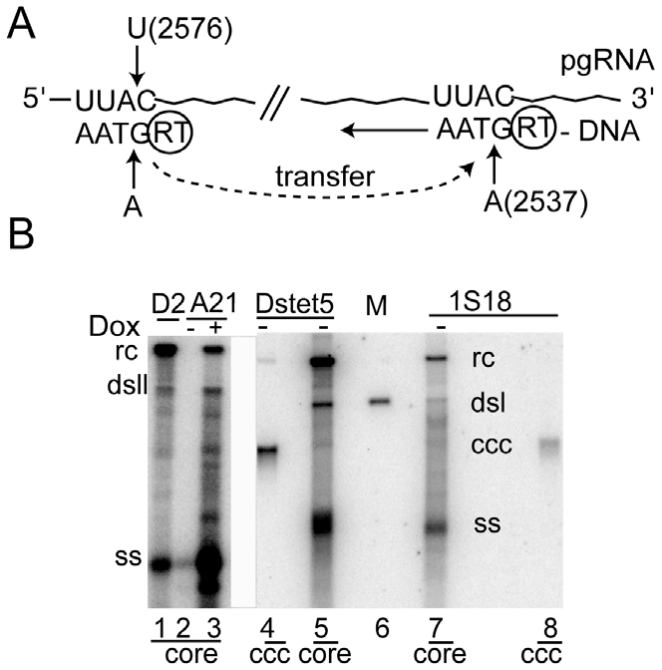
Cell lines expressing DHBV mutant C2537T. A. The figure shows the first steps in reverse transcription of pregenomic (pg) RNA (for a detailed description see ref. [3]). DHBV mutant C2576T leads to the synthesis of minus strands with four nucleotides beginning with dA in place of dG. After the transfer of the RT-DNA complex to the 3′ end of pregenomic RNA, the primer anneals with complementary sequences spanning positions 2534–2537. As a consequence of this reaction, the mutation introduced at position 2576 appears on position 2537 of RC and CCC DNA. B. The figure depicts DHBV DNA isolated from intracellular core particles (core, lanes 1–3, 5 and 7)) and CCC DNA (ccc, lanes 4 and 8) expressed in D2, A21, Dstet5 and 1S18 cells. A21 and 1S18 cells express mutant DHBV mutant C2576T. Dstet5 and 1S18 cells express DHBV without envelope proteins, and A21 and 1S18 cells express DHBV in the presence and absence of doxycycline (dox), respectively. Rc; relaxed circular, dsl; double strand linear, ss; single stranded, ccc; covalently closed circular. M; linear DHBV genome (1 ng) released from plasmid DNA (lane 6).

**Figure 3:**
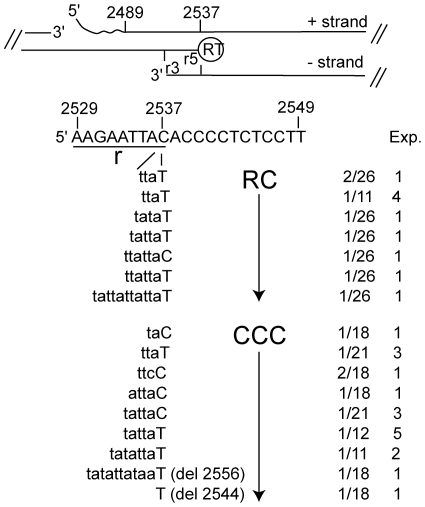
RC and CCC DNA sequence analysis. The upper part of the figure shows the positions of the 5′ ends of plus (pos. 2489) and minus strand DNA (pos. 2537) attached to RNA (waved line) and RT, respectively (nucleotide positions according to K01834). The positions of the r regions are indicated (r5, r3). The lower part of the figure shows the nucleotide sequence of plus strands between positions 2529–2549 including the r region (pos. 2529–2537). The sequence of cloned PCR fragments derived from RC and CCC DNA with insertions between position 2536 and 2537 are shown in lower case together with the sequence at position 2537 in upper case. The last two sequences were derived from two clones with deletions spanning positions 2538–2556 and 2544, respectively (see also Tables 1 and 2).

A distinction between the two models for CCC DNA formation requires information about the fate of the small redundant sequences r5 and r3 on minus strands of RC DNA. Model 1 predicts that r5 is present in CCC DNA whereas model 2 predicts that r5 is removed prior to CCC DNA formation (Figure 1B,C). To determine the origin of the r region in CCC DNA, we took advantage of detailed information about the mechanism for reverse transcription of minus strand DNA (Figure 2A and [3]). Priming of minus strand DNA synthesis occurs from a tyrosine residue in the N-terminal domain of the RT [16]. The template for this reaction is a C residue located near the 5′ end of pregenomic RNA within the RNA packaging signal (Figure 2A, pos. 2576) [17]. Following the formation of the RT-dGMP bond and the synthesis of three additional nucleotides, the nascent DNA strand transfers to position 2537 near the 3′end of the viral RNA where minus strand DNA synthesis continues [17], [18]. To genetically tag the r5 region, we have mutated nucleotide 2576 from dC to dT. Accordingly, this mutation changes the first nucleotide of minus strand DNA from dG to dA (Figure 2A). As a consequence, r5 differs from r3 by one nucleotide at position 2537 corresponding to the first nucleotide of minus strand DNA. Depending on the mechanism for the conversion of RC into CCC DNA, CCC DNA should either carry a G:C or an A:T bp at position 2537. However, the frequency by which either base pair appears on CCC DNA also depends on the mechanism for plus strand DNA synthesis, which requires that nascent plus strands switch templates from r5 to r3 (Figure 1A). To draw firm conclusions about the origin of the nucleotide at position 2537 in CCC DNA, the frequency by which nascent plus strands are extended to position 2537 in r5 prior to the template switch must be known.

### Establishment of Cell Lines

To perform our investigations, we established two cell lines, A21 and 1S18, permitting the conditional expression of DHBV genomes carrying the C2576T mutation. DHBV genomes expressed in 1S18 cells are defective in the production of envelope proteins and as a consequence accumulate higher levels of CCC DNA than A21 cells [19], facilitating the analysis of CCC DNA as described below. Both cell lines expressed the major DNA replication intermediates characteristic of DHBV replication following activation of the tet transactivator through addition (A21) or removal (1S18) of doxycycline from the culture media (Figure 2B). These results indicated that introduction of the C2576T mutation did not interfere with viral DNA replication.

### Analysis of Plus Strand RC DNA

To determine the nucleotide sequence at position 2537 on plus strand DNA, we PCR amplified two regions in RC DNA spanning positions 2507 to 2760 and 2477 to 2760, respectively. The primers annealed to the 5′ end of plus strand DNA on either side of the discontinuity in minus strand DNA favoring amplification of plus over minus strands (Figure 3). RC DNA was obtained from concentrated virus secreted by A21 cells and from intracellular core particles present in 1S18 cells. Nucleotide sequence analysis of 26 cloned fragments derived from virion DNA secreted in A21 cells yielded four types of sequences: wildtype (8%), wildtype with an insertion at position 2537 (4%), mutant C2537T (70%) and C2537T with insertions at position 2537 (17%) (Figure 3). Insertions ranged from 3–11 nucleotides in length and represented repeats corresponding to the first three or four nucleotides of minus strand DNA. Similar results were obtained with three additional experiments with core particle-derived RC DNA, except that we did not observe any clones with insertions (Table 1). Thus, the presence of the C2537T mutation varied between the four independent experiments from 82% to 88% and occurred with an average of 85%.

**Table 1:** Sequence analysis of RC DNA.

*Experiment*	*1 (n = 26)* 1,3	*2 (n = 11)* 1	*3 (n = 24)* 1	*4 (n = 11)* 2	*5 (n = 10)* 2,4
***Wildtype***	2 (8%)	2 (18%)	3 (13%)	2 (18%)	6 (60%)
***Wildtype ins***	1 (4%)	0	0	0	0
***C2537T***	18 (70%)	9 (82%)	21 (88%)	9 (82%)	3 (30%)
***C2537T ins***	5 (17%)	0	0	0	1 (10%)
***C2537T total***	23 (87%)	9 (82%)	21 (88%)	9 (82%)	4 (40%)

1 primers 2507, -2760.

2 primers 2477, -2760.

3 virion DNA, A21 cells.

4 DNA was treated with NaOH to remove RNA primer.

To verify that plus strands were indeed the primary template for the PCR reactions, we incubated core DNA with sodium hydroxide to remove the RNA primer attached to the 5′ end of plus strand DNA, thus removing 12 nts of the annealing site for primer 2477 during the PCR reaction (Table 1, experiment 5, Figure 3). Those conditions favored amplification of minus strand DNA over the gap region, which is facilitated by the presence of the r region. Moreover, nascent DNA strands reaching the first nucleotide of minus strand at position 2537 are not extended with high efficiency due to the mismatch that occurs with r3 following the required template switch. As a result the fraction of clones with wildtype sequences should be larger than observed under normal conditions (experiments 1–4). The results obtained with experiment 5 were consistent with this prediction. Only 40% of the clones analyzed carried the C2537T mutation (Table 1). Chi-square analysis revealed a significant difference between experiments 1–4 and 5 (p = 0.0027).

The insertion mutations could have occurred by two mechanisms. First, the insertions could have been caused by a defect in the priming reaction for minus strand DNA synthesis due to the C2576T mutation. This possibility is favored by observations made with similar mutations in HBV and DHBV, respectively that led to the synthesis of minus strands with extra nucleotides at their 5′ ends ([20] and D. Loeb, personal communication). It appears that mutations altering the initiation site for minus strand synthesis interfere with the proper transfer of the nascent DNA strand to the 3′ end of pregenomic RNA and, as a consequence, induce one or more “slippage-initiation” cycles before the transfer finally occurs. Alternatively, we cannot completely rule out that at least some of the insertions occurred during the transfer of plus strand DNA synthesis due to the mismatch created by the sequence divergence between r5 and r3 at position 2576. Finally, it should be noted that insertions were not detected in clones derived from RC DNA of wildtype virus, confirming that they were not created during the PCR reaction (results not shown).

In summary, these experiments demonstrated that in more than 80% of events, the viral polymerase extended nascent plus strands until it reached the first nucleotide of minus strand DNA at position 2537. Loeb and colleagues arrived at a similar conclusions from their experiments with DHBV genomes that carried mutations in r3 [21].

### Analysis of CCC DNA

The analysis of RC DNA revealed that on average 85% of plus strands carry the C2576T mutation (Table 1). Model 1 predicts that all CCC DNA molecules receive the mutation from the 5′ end of minus strand and 85% from plus strand DNA. Fifteen percent of CCC DNA molecules require DNA mismatch repair. Thus, model 1 predicts that the fraction of CCC DNA molecules with the C2576T mutation could range from 85–100% (average 93%) depending on which DNA strand is used as the template for DNA mismatch repair (Figure 1B, model 1). In contrast, model 2 predicts that all CCC DNA molecules receive wildtype sequences from minus strand DNA and 85% the mutation from plus strands. Hence, the C2576T mutation should occur with a theoretical frequency ranging from 0–85% (average 43%) depending on the template preference for the DNA repair reaction (Figure 1C).

To determine the genotype at position 2537 in CCC DNA, we isolated CCC DNA from 1S18 cells. Because the concentration of RC DNA is much higher than CCC DNA, CCC DNA preparations can be contaminated with RC DNA even when methods are used that favor isolation of CCC over RC DNA. To further reduce the amount of contaminating RC DNA, we took advantage of differences in the migration of the two DNA forms in agarose gels and used gel purified CCC DNA for two of five experiments (Table 2). The resulting DNA fractions were free of detectable RC DNA (results not shown). Like with RC DNA, we used two primer pairs spanning positions 2507 to 2760 and 2429 to 2605, respectively to determine the nucleotide at position 2537. With the first primer pair, the C2537T mutation occurred with a frequency of 22%, 57% and 64%, respectively (Table 2). With the second pair, the frequency was 64% in two independent experiments. Like with RC DNA, eight clones derived from CCC DNA carried insertions. In addition, two clones exhibited deletions adjacent to position 2537 (Figure 3). Most likely, the deletions were the result of DNA repair reactions that occurred during or following CCC DNA formation. Unexpectedly, the lowest frequency of mutations was observed with CCC DNA that was not gel purified (experiment 1, Table 2). This experiment also yielded the largest number of clones with insertions that exhibited (wildtype) dC at position 2537.

**Table 2:** Sequence analysis of CCC DNA.

*Experiment*	*1 (n = 18)* 1	*2 (n = 11)* 2,3	*3 (n = 21)* 1,3	*4 (n = 11)* 1	*5 (n = 11)* 2
***Wildtype***	10 (56%)	4 (36%)	8 (38%)	4 (36%)	4 (36%)
***Wildtype ins***	4 (22%)	0	1 (5%)	0	0
***C2537T***	2 (11%)	6 (55%)	11 (52%)	7 (64%)	6 (55%)
***C2537T ins***	0	1 (9%)	1 (5%)	0	1 (9%)
***C2537T del***	2 (11%)	0	0	0	0
***C2537T total***	4 (22%)	7 (64%)	12 (57%)	7 (64%)	7 (64%)

1 primers 2507, -2760.

2 primers 2429, -2605.

3 DNA was gel-purified.

The results obtained with CCC DNA revealed that in all five experiments the occurrence of the C2537T mutation was less than 85%, below the frequency ranging from 85–100% (average 93%) predicted by model 1 (Figure 1B). The mutation occurred in four of five experiments with a higher frequency than 43% expected from model 2 assuming random mismatch repair. This result indicated that mismatch repair was not completely random and favored minus over plus strand DNA for the selection of the template for the repair reaction. Moreover, it is conceivable that we underestimated the frequency of the mutation in RC DNA due to aberrant amplification of r3 as it occurred under the conditions used for experiment 5 (Table 1).

To validate our favoring model 2 we asked: ‘If the chance of mutation is 80% how likely is it that we would observe only 37 mutants in 72 observations, when we would expect to see about 58 (0/8*72 = 57.6)?’ This chance turns out to be less than 2 in 10 million, and led us to reject the possibility that the mutation frequency is at least 80%. Even if we omitted the results from experiment 1, which yielded the highest percentage of wildtype clones, the same method shows that the chance of seeing only 33 mutants in 54 observations is less than 2 out of 1000. Thus, based on statistical analyses we concluded that our results supported model 2.

### Implications for HBV Replication

Our results demonstrated that the r5 region of minus strand DNA is completely or at least partially removed prior to CCC DNA synthesis and as a consequence that the r region in CCC DNA must be derived from r3 or from a combination of r3 and r5. These results were inconsistent with a model predicting a catalytic function of the RT to cleave the phosphodiester bond defining the 5′ end of r3 and ligate it with the dGMP attached to tyrosine in a cleavage-ligation reaction. In contrast, the results were consistent with a detailed genetic analysis of the DHBV RT, which failed to reveal mutants with specific defects in CCC DNA synthesis [22]. However, the detection of such mutants could have been obscured by an overlapping function on the RT required for RC DNA synthesis. Nevertheless, we interpreted our results to mean that both RC DNA strands undergo DNA repair reactions mediated by cellular enzymes once the DNA is delivered into the cell nucleus.

Recent analyses of viral DNA replication intermediates revealed a protein-free RC DNA, termed PF-RC DNA [14], [15]. PF-RC DNA contains minus strands with 5′ ends that are identical with the natural 5′ ends of minus stand DNA created during reverse transcription of pregenomic RNA. A follow-up study provided evidence for a role of a serine protease activity in the removal of the RT from RC DNA, suggesting that PF-RC DNA still contains residual amino acids derived from the RT covalently attached to its 5′ end [23]. Our results are consistent with the possibility that PF-RC DNA represents an intermediate in CCC DNA formation that requires further processing of the 5′ ends of minus strand DNA by an endonuclease as described below.

**Figure 4:**
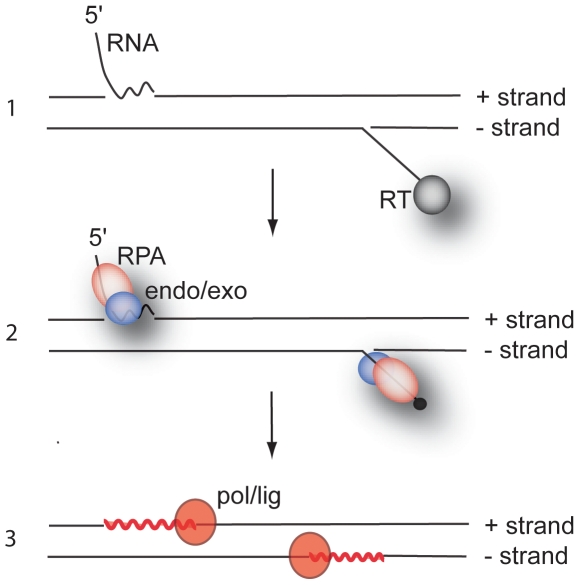
Mechanism proposed for the formation of CCC DNA. The figure depicts the 5′ ends of plus and minus strand DNA of RC DNA linked to RNA and RT, respectively (1). Endo/exo refers to the endonucleases Fen1 and DNA2 that might be recruited by RPA to the single stranded regions (see text) (2). Pol/lig refer to the DNA polymerase and ligase required for the DNA repair reactions (zig-zagged lines) leading to the formation of CCC DNA (3).

How can we envisage the sequence of events that lead to CCC DNA formation? One possibility is that the 5′ ends of RC DNA signal the presence of two Okazaki fragments and recruit the lagging strand machinery for the maturation step leading to CCC DNA formation. In this model the single-stranded DNA binding protein replication protein-A (RPA) might bind to the 5′ ends and recruit the endonucleases Dna2 or Fen1 and the exonuclease Exo1 to cleave the RNA primer at the 5′ end of plus strands (Figure 4, [24], [25]). DNA polymerase δ or ε might then extend the 3′ ends, followed by a DNA ligation reaction to join the ends. The completion of minus strands would proceed in the same fashion except that an endonuclease would have to cleave the 5′ end of minus strand distal to r5. Whether Dna2 or Fen1 could cleave a “flap” attached to protein rather than RNA is not known. But, based on the recent reports described above, it is possible that the substrate for CCC DNA formation contains only a few amino acids at the 5′ end of minus strand DNA, which might not interfere with the activities of these enzymes. Identification of the cellular components required for CCC DNA formation will be the next step for a better understanding of the hepadnavirus life cycle and depend on the development of an *in vitro* system that can recapitulate the enzymatic reactions necessary for the conversion of RC into CCC DNA.

## Materials and Methods

### Plasmids

The plasmid puctetDHBVC2576T was derived from puctetDHBV by site-directed mutagenesis of C to T at nucleotide position 2576 [26], [27]. The DHBV pregenomic (pg) RNA is transcribed under the control of CMV-tet promoter. Plasmid pDHBVC2576T1S was constructed by replacing a BglII and BstEII fragment with the corresponding segment from pDHBV1S containing three stop codons in the envelope protein [26].

### Cell Lines

The chicken hepatoma cell line LMH-derived A21 and 1S18 cell lines were maintained in Dulbecco's modified Eagle medium-F12 containing 10% fetal bovine serum, 200 ug/ml G418, and 1 ug/ml doxycycline or tetracycline (1S18) [28]. To establish the A21 cell line, LMH cells were transfected with plasmids puctetDHBVC2576T, pUHrT62-1 (tet-on), and pRSVneo at a ratio of 5∶5∶1, respectively. For the 1S18 cell line, LMH cells were transfected with pDHBVC2576T1S, pUHD15.1 (tet-off), and pRSVneo. The cells lines were selected in medium containing G418 at 400 ug/ml, for 1S18, in the presence of doxycycline (1 µg/ml).

### Extraction of Viral DNA

To induce DNA replication for the analysis of RC and CCC DNA, A21 and 1S18 cells were cultured with (A21) or without (1S18) doxycycline for six days. For the isolation of CCC DNA, we used a method developed by Summers and colleagues [29]. Briefly, cell monolayers on 10 cm plates were treated with 2.4 ml of ice-cold cell lysis buffer (5 mM Tris:Hcl (pH 7.5), 1 mM EDTA (pH 8.0), and 0.2% NonidetP-40). An equal volume of alkali lysis buffer (0.1N NaOH, 6% SDS) was added and the mixture was incubated for 30 minutes at 37°C, neutralized with 3M potassium acetate (pH 5.0) to a final concentration of 0.6 M and centrifuged at 12,000 rpm for 5 minutes. The supernatant was extracted twice with phenol followed by extraction with butanol:isopropanol (7∶3) to remove residual phenol. The DNA was then precipitated with 2.2 volumes of ethanol for 20 minutes at −70°C. As indicated, CCC DNA was gel purified by electrophoresis through 1.5% agarose and recovered using Qiaquik gel extraction kit.

For isolation of core DNA, one 10 cm plate of cells was treated with 2.4 ml of ice-cold cell lysis buffer (10 mM Tris-HCl (pH 8.0), 1 mM EDTA, 1.0% NP-40) and centrifuged at 13,000 rpm. The supernatant was precipitated with 0.25 volumes of 35% PEG-8000 in 1.75NaCl, incubated on ice for 30 minutes, and centrifuged at 8,000 rpm for 10 minutes. The resulting pellet was dissolved in the proteinase K buffer (10 mM Tris-HCl (pH 8.0), 100 mM NaCl, 1 mM EDTA, 0.5% SDS, 200 ug/ml proteinase K) and incubated for one hour at 45°C. The core DNA was then phenol extracted once, butanol:isopropanol extracted, and ethanol precipitated as for CCC DNA isolation.

For the isolation of virion DNA from A21 cells, supernatant was cleared from cellular debris and virus concentrated by ultracentrifugation in an SW40 rotor at 39,000 rpm for 35 minutes. Pellets were dissolved in proteinase K buffer containing tRNA and purified as described for core DNA.

### PCR Amplification

PCR amplifications were carried out for 25 to 30 cycles with Advantage 2 *Taq* DNA polymerase (Clontech) using the following primer sets: 2477 (pos. 2477–2497; 5′ TAC ACC CCT CTC TCG AAA GC 3′) and 2760 (pos. 2760–2740; 5′ CCA ATA AGG CTC TAA AGC GTC 3′); 2507 ( pos. 2507 to 2527, 5′ CCA CAT AGG CTA TGT GGA ACT 3′) and 2760; 2429 (pos. 2429 to 2444, 5′ GCT GAC GGC CCA TCC A 3′) and 2605 (pos. 2605 to 2583, 5′ CAG TCA CAC ACG ACA ACA GCA AT 3′). For sequence analysis all PCR fragments were cloned into pGEMT-Easy (Promega) and sequenced with primers annealing to the T7 or SP6 promoters. The numbering of the nucleotide positions is according to ref. [27] (Genbank accession number K01834).
